# Biocompatible and flexible paper-based metal electrode for potentiometric wearable wireless biosensing

**DOI:** 10.1080/14686996.2020.1777463

**Published:** 2020-06-24

**Authors:** Toshiya Sakata, Masami Hagio, Akiko Saito, Yuto Mori, Masayuki Nakao, Kazuhiko Nishi

**Affiliations:** a Department of Materials Engineering, School of Engineering, the University of Tokyo, Tokyo, Japan; b Department of Mechanical Engineering, School of Engineering, the University of Tokyo, Tokyo, Japan

**Keywords:** Paper-based electrode, potentiometric measurement, bioelectrical interface, wireless, wearable biosensor, 208 Sensors and actuators, 212 Surface and interfaces

## Abstract

A paper-based electrode is a very attractive component for a disposable, nontoxic, and flexible biosensor. In particular, wearable biosensors, which have recently been attracting interest, not only require these characteristics of paper-based electrodes but must also be able to detect various ions and biomolecules in biological fluids. In this paper, we demonstrate the detection ability of paper-based metal electrodes for wearable biosensors as part of a wireless potentiometric measurement system, focusing on the detection of pH and sodium ions. The paper-based metal electrodes were obtained by simply coating a silicone-rubber-coated paper sheet with a Au (/Cr) thin film by sputtering then modifying it with different functional membranes such as an oxide membrane (Ta_2_O_5_) and a fluoropolysilicone (FPS)-based Na^+^-sensitive membrane, corresponding to the targeted ions. Satisfactory and stable detection sensitivities of the modified paper-based Au electrodes were obtained over several weeks even when they were bent to a radius of curvature in the range of 6.5 to 25 mm, assuming use in a flexible body patch biosensor. Moreover, the Na^+^ concentration in a sweat sample was evaluated using the paper-based Au electrode with the FPS-based Na^+^-sensitive membrane in a wireless and real-time manner while the electrode was bent. Thus, owing to their complex mesh structure, flexible paper sheets should be suitable for use as potentiometric electrodes for wearable wireless biosensors.

## Graphical abstarct

**Figure:**
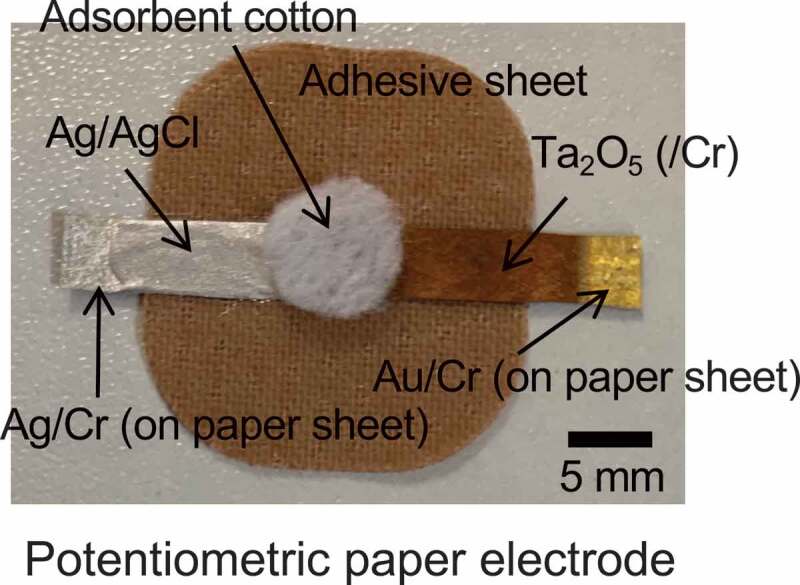

## Introduction

Wearable biosensors have recently been attracting interest for health monitoring [1–3]. Various types of biosensor are integrated to simultaneously monitor ions and biomolecules in biological fluids, such as sweat on a flexible substrate, in a wireless manner. Using such wearable biosensors, we can monitor our health noninvasively in real time without collecting blood samples with needles. The obtained data of an individual should be accumulated and analyzed in an electronic device connected to the internet so that it can be easily shared with her/his family and doctors as well as the person in question as part of health checks and for the early diagnosis of diseases. Furthermore, flexible and nontoxic electrodes are required for wearable devices applied as patches on a body. Thus, paper-based electrodes can potentially be used in wearable biosensors.

Lateral flow immunochromatographic assays, which are carried out using a cellulose-based device, are suitable as part of a low-cost and simple system to detect target analytes [4,5]. Biological sample solutions easily flow through a paper sheet, and then biomolecular recognition events such as an antigen–antibody reaction are colorimetrically detected on the sheet without specialized equipment, where antibody-conjugated tags such as gold nanoparticles are included and reacted with the antigen at the test line. However, this measurement method is not quantitative and cannot continuously monitor vital signs. Therefore, if a paper sheet is developed as an electrode, which will enable the real-time measurement of electrical signals, the advantages of paper such as flexibility and nontoxicity will contribute to biological sensing by wearable devices. In previous works, paper-based electrodes were developed for the detection of biomolecules such as glucose and cortisol. Most of them employed amperometric and impedimetric methods combined with enzymes and redox mediators [6–12]. On the other hand, the modification of ion-sensitive membranes (ISMs) on a paper-based electrode will be useful for the detection of ions in biological fluids, such as sweat, as part of a potentiometric measurement system.

ISMs have been widely used in potentiometric biosensors for several decades [1,13–20]. When a solution-gate field-effect transistor (FET) is utilized as an ion sensor, oxide membranes (e.g., Ta_2_O_5_) as the gate insulator, which behave as ISMs, have hydroxy groups at their surfaces in a solution that undergo an equilibrium reaction with hydrogen ions [15,17,20]. As a result, a change in the interfacial potential between the solution and the oxide membrane surface is output as the Nernstian response for the change in pH, which is ideally calculated as 59.2 mV/pH at 25°C. Cellular respiration activities were monitored at a cell/gate nanogap interface using a pH-sensitive FET in a real-time and noninvasive manner [21–27]. When polyvinyl chloride (PVC)-based ISMs with ionophores are coated on a gate insulator, the Nernstian response is similarly obtained for the change in the potential at the solution/ISM interface with ions of various concentrations (e.g., Na^+^ and K^+^) [1,16,18,19]. However, these PVC-based ISMs include a plasticizer, which exhibits cytotoxicity, to dissolve ionophores in the membrane. Therefore, a fluoropolysilicone (FPS)-based plasticizer-free ISM has been proposed for use as a patch biosensor on bodies[28]. Thus, oxide membranes and FPS-based plasticizer-free ISMs on paper-based electrodes can be modified to realize biocompatible wearable biosensors.

In this study, we demonstrated the detection ability of paper-based metal electrodes for use in wearable biosensors as part of a wireless potentiometric measurement system, focusing on the detection of hydrogen ions and sodium ions. In particular, nontoxic materials were chosen for the paper-based metal electrode of a patch biosensor placed on the skin. Here, we utilized a silicone-rubber-coated paper sheet as the paper-based metal electrode to smoothly coat a Au or Ag (/Cr) thin film by sputtering.

## Methods

### Materials

The following chemicals and materials were used in this study. Tetrakis[3,5-bis(trifluoromethyl)phenyl]borate sodium salt (Na-TFPB), 4-(2-hydroxyethyl)-1-piperazineethanesulfonic acid (HEPES), and tetrahydrofuran (THF) were purchased from Dojindo Molecular Technologies Inc. 4-Tert-butylcalix[4]arene-tetraacetic acid tetraethyl ester (calix[4]arene) was purchased from Sigma Aldrich. Sodium dihydrogen phosphate (NaH_2_PO_4_), sodium hydrogen phosphate (Na_2_HPO_4_), citric acid, potassium chloride (KCl), sodium hydroxide (NaOH), phosphate-buffered saline (PBS), and pH standard buffer solutions were purchased from Wako Pure Chemicals Industries, Ltd. Fluorosilicone gel (FE-73) and 3-aminopropyltriethoxysilane (APTES) were purchased from Shin-Etsu Chemical Co., Ltd. Paper sheets, both sides of which were coated with silicone rubber, were purchased from Asahi Kasei Home Products Corporation.

### Fabrication of paper-based metal electrodes

The paper-based metal electrodes used in this study were fabricated on flexible silicone-rubber-coated paper sheets (35 mm × 5 mm) by sputtering (ULVAC, Inc.). First, Cr (thickness 15 nm) was sputtered as an adhesive layer. Then, a Au or Ag thin film (thickness 100 nm) was sputtered. We call it the paper-based metal electrode in this study. Moreover, tantalum oxide (Ta_2_O_5_) was coated to a thickness of 100 nm on the paper-based Au electrodes (5 mm × 5 mm) to obtain a Ta_2_O_5_ membrane with pH-responsive hydroxyl groups at its surface in a solution (Figure S1, Supporting Information). The surface morphology of the paper-based Au electrodes with or without surface modifications was observed by scanning electron microscopy (SEM).

### Preparation of plasticizer-free Na^+^-sensitive membrane

A plasticizer-free Na^+^-sensitive membrane was prepared as follows. After mixing 4.5 mg of calix[4]arene as an ionophore, 2.0 mg of Na-TFPB as an agent for anion exclusion, and 150 mg of FE-73 as a base material in 750 μL of THF, 10 μL of the mixed solvent was added dropwise to the Ta_2_O_5_ film surface and then heated at 120°C for 2 h in ambient air. The heat treatment induced the addition reaction of hydrosilylation for polymerization[28].

### Wireless and miniaturized measurement system

**Figure 1.**
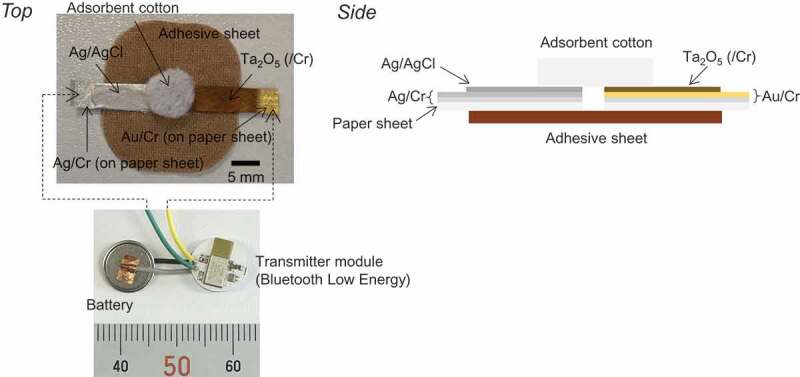
Paper-based metal electrode and electrical measurement system for wireless sensing.

Figure 1 shows photographs of a wireless device with paper-based metal electrodes. As a reference electrode, a Ag/AgCl ink was pasted on the paper-based electrode with a Ag thin film and then heated at 120°C for 2 h in ambient air. In the measurement, adsorbent cotton (diameter 5–10 mm) was placed on both the working electrode and the reference electrode to retain sample solutions. 100 μL of the sample solutions was added dropwise to the adsorbent cotton on both electrodes. Each type of paper-based metal electrode was connected to a wireless interfacial potential detector (IPD), and the change in the interfacial potential between the solution and the electrode surface (μ*V*_out_) was measured with a real-time and wireless monitoring system developed by IoT Media Laboratory (Department of Mechanical Engineering, The University of Tokyo). We developed the wireless IPD, which was composed of MCP3425 (16 bit ADC; Microchip Technology Inc.), EYSHSNZWZ (ultra-small BLE Arm Processor Module; Taiyo Yuden Co., Ltd.) as a wireless module, and a small and thin coin type lithium battery (3.7 V; Maxell Holdings, Ltd.). μ*V*_out_ was measured in a solution through the reference electrode (Ag/AgCl) with high accuracy (16 bit resolution). The wireless module (EYSHSNZWZ) was newly programmed in the above measurement by IoT Media Laboratory (Department of Mechanical Engineering, The University of Tokyo). To investigate pH responsivity, phosphate buffer solutions were prepared with pHs from 5.81 to 8.11 by controlling the mixing ratio of Na_2_HPO_4_ to NaH_2_PO_4_. Also, standard buffer solutions with pHs of 4.01, 6.86, 7.41, 9.18, and 10.01 were prepared. Moreover, the concentration of Na^+^ was varied from 5 mM to 1 M by adding an appropriate volume of 1 M NaCl into a mixed solution, which was composed of 50 mM HEPES and 1 M NaOH in deionized water, to maintain a constant pH of 7.2.

### In vitro *and on-body analysis of Na^+^ concentration in sweat sample*

A sweat sample was collected from one healthy volunteer in a sauna for *in vitro* real-time monitoring of the sweat sample. Firstly, the actual concentration of Na^+^ in the sweat sample was measured using the conventional Na^+^ sensor (LAQUAtwin-Na-11; HORIBA, Ltd.). On the other hand, μ*V*_out_ was monitored with changing Na^+^ concentrations (5 mM → 10 mM → 50 mM → 100 mM → 500 mM) using the paper-based Au electrode with the FPS-based Na^+^-sensitive membrane in a wireless manner. Then, the sweat sample was also measured during these measurements, considering the actual concentration of Na^+^. The sample volume added was 100 μL and the paper-based Au electrodes with the FPS-based Na^+^-sensitive membrane were prewashed before adding each sample solution. Moreover, the paper-based Au electrode with the FPS-based Na^+^-sensitive membrane was put on the forehead to monitor the Na^+^ concentration in a wireless and real-time manner. The on-body measurement was performed on the same healthy volunteer in the sauna for about 40 minutes on another day. Then, the sweat samples were collected every 100 seconds at a certain interval to confirm the actual Na^+^ concentrations using the conventional Na^+^ sensor. The effect of the temperature during measurements on electrical signals was compensated for considering the data measured inside and outside the sauna.

## Results and discussion

### Electrical characteristics

**Figure 2.**
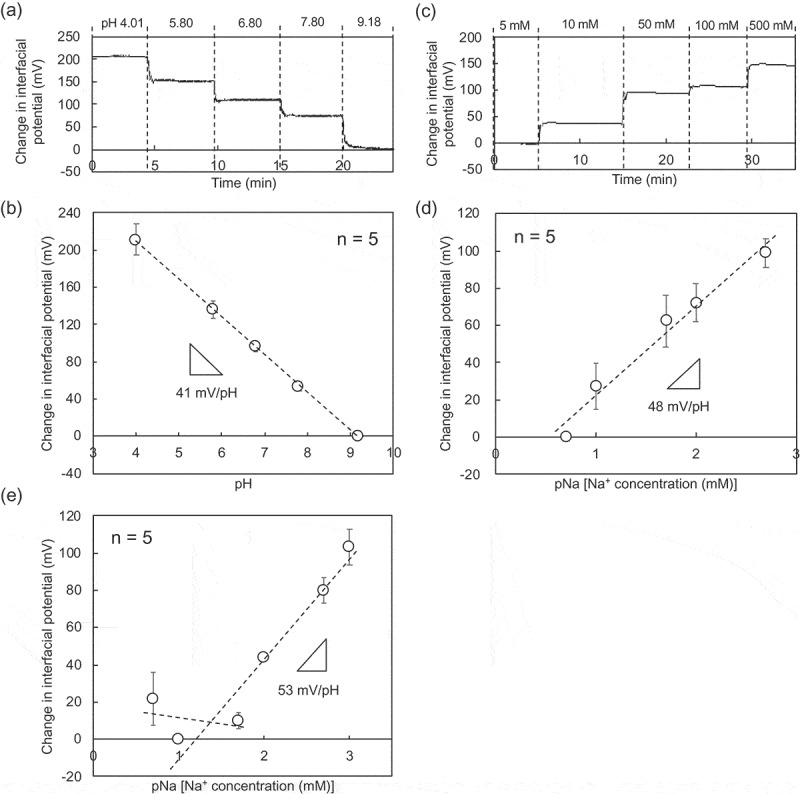
Electrical characteristic of paper-based metal electrodes without bending obtained in wireless manner. (a) Change in interfacial potential with pH from 4.01 to 9.18 using the paper-based Au electrode with the Ta_2_O_5_ film. (b) Calibration curve for pH sensitivity based on (a). The number of measurements was five. (c) Change in interfacial potential with Na^+^ concentration from 5 to 500 mM detected using the paper-based Au electrode with the FPS-based Na^+^-sensitive membrane. (d) Calibration curve for pNa sensitivity based on (c). The number of measurements was five. (e) pNa response of the paper-based Au electrode with the FPS-based Na^+^-sensitive membrane in a buffer with 100 mM K^+^. In (a) and (b), the interfacial potentials at pH 9.18 were offset to zero. In (c) and (d), the interfacial potentials at 5 mM Na^+^ were offset to zero. In (e), the interfacial potential at 5 mM Na^+^ and 100 mM K^+^ was offset to zero.

The electrical characteristics of paper-based metal electrodes were first investigated without bending the electrodes. Figure 2(a,b) show μ*V*_out_ for different pHs using the paper-based Au electrode with the Ta_2_O_5_ film. The calibration curve was analyzed on the basis of the data obtained by the wireless and real-time measurement. The pH sensitivity was calculated to be about 41 mV/pH. In general, the Nernstian response to the change in H^+^ concentration is generally expressed as

(1) $$\Delta E=59.2\times \Delta \;log\;\left[H^{+}\right]\;\left(mV/decade\right)\;25^{\circ }C.\;$$

On the other hand, $\psi_{0}$ is shown to be the actual driving force of an ion-sensitive field-effect transistor (ISFET) with the Ta_2_O_5_ membrane, which is related to the logarithm of H^+^ concentration by the following equations[29]:

(2) $$\psi_{0}=2.303\frac{kT}{q}\left(\frac{\beta }{\beta +1}\right)log\left[H^{+}\right]$$

(3) $$\beta =\frac{2q^{2}N_{S}{\left(K_{a}K_{b}\right)}^{1/2}}{kTC_{DL}}$$

where *k* is the Boltzmann constant, *T* is the absolute temperature, *q* is the elementary charge, and *C_DL_* is the capacitance of the electric double layer. The parameter β reflects the chemical sensitivity of the Ta_2_O_5_ membrane, which depends on the site density $N_{S}$ of the hydroxy groups at the surface, and the surface reactivity is characterized by the equilibrium constants $K_{a}$ and $K_{b}$ of the acidic and basic surface reactions, respectively. If the parameter β is much higher for the Ta_2_O_5_ membrane, which results from a higher $N_{S}$, the difference in $\psi_{0}$ for μpH ($\Delta \psi_{0}$) determined using Equation (2) should be almost equal to that determined using Equation (1), that is, the Nernstian response. Otherwise, $\Delta \psi_{0}$ would not be satisfied with the Nernstian response. Thus, if the Ta_2_O_5_ membrane is fabricated on a substrate by sputtering, the surface property of the Ta_2_O_5_ membrane in contact with a solution may be different, depending on the material used as a substrate. In this study, the Ta_2_O_5_ membrane was coated on the paper-based Au electrode by sputtering; the Nernstian response was not sufficiently attained as 41 mV/pH, which means that $N_{S}$ would have been lower. On the other hand, the Ta_2_O_5_ membrane on oxide-film-coated substrates, which was fabricated using the sputtering system, contributed to the Nernstian response (approximately 52–56 mV/pH) [28,30]. Moreover, the paper-based Au electrode with the FPS-based Na^+^-sensitive membrane exhibited a pNa sensitivity of about 48 mV/pNa, as shown in Figure 2(c,d). In particular, the selective detection of Na^+^ of various concentrations was realized using the paper-based Au electrode with the FPS-based Na^+^-sensitive membrane, as shown in Figure 2(e). In this case, the Na^+^ concentration was changed from 5 mM to 1 M under a constant K^+^ concentration of 100 mM. The selectivity coefficient $K_{Na^{+},K^{+}}$ for Na^+^ under the constant K^+^ concentration was calculated to be about 0.23 from equations S1 and S2 (Supporting Information), which means that the pNa sensitivity remained at about 53 mV/pNa above a Na^+^ concentration of about 23 mM. This means that a change in the K^+^ concentration of less than 100 mM would have almost negligible effect on the pNa sensitivity above a Na^+^ concentration of 20 mM. In fact, the K^+^ concentration in human perspiration is in the range of 1–10 mM, whereas the Na^+^ concentration is in the range of 30–80 mM [31–34]. That is, the change in the Na^+^ concentration in secreted sweat can be selectively detected using the paper-based Au electrode with the FPS-based Na^+^-sensitive membrane.

**Figure 3.**
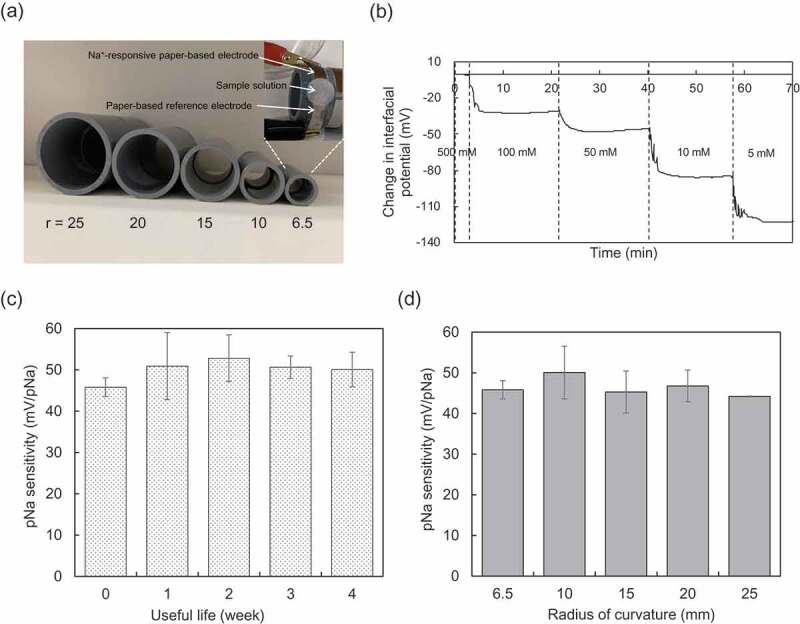
Electrical characteristic of paper-based metal electrode with bending obtained in wireless manner. (a) Photograph of polyvinylchloride resin-based cylinders with different radii (*r*) from 6.5 to 25 mm. The paper-based Au electrode with the FPS-based Na^+^-sensitive membrane, which was used in (b), (c), and (d), was rolled around the cylinder with the smallest radius of 6.5 mm (upper-right photograph). (b) Real-time monitoring of μ*V*_out_ using the paper-based Au electrode with the FPS-based Na^+^-sensitive membrane, which was rolled around the cylinder with the smallest radius of 6.5 mm (at useful life 0 week). (c) Useful life of the paper-based Au electrode with the FPS-based Na^+^-sensitive membrane, which was rolled around the cylinder with the smallest radius of 6.5 mm. The change in pNa sensitivity was evaluated for 0 to 4 weeks. The number of measurements performed at each time was three. (d) Change in pNa sensitivity with radius of curvature from 6.5 to 25 mm detected using the paper-based Au electrodes with the FPS-based Na^+^-sensitive membrane, which were rolled around each cylinder.

Next, the effect of the bending of the paper-based metal electrodes on the electrical properties was examined, assuming their use in a device on the skin. Here, the paper-based metal electrodes were rolled around cylinders with radii from 6.5 to 25 mm (Figure 3(a)). Figure 3(b,c) show the time course of μ*V*_out_ for different pNa and the long-term stability of the pNa sensitivity using the modified paper electrode with the radius of curvature of 6.5 mm, respectively. The electrical stability, responsivity, and sensitivity were maintained even at the smallest radius of curvature, regardless of whether or not the paper-based metal electrodes were bent. Moreover, the pNa sensitivity was maintained at around 50 mV/pNa, near the Nernstian response, regardless of the radius of curvature (Figure 3(d)). Thus, the paper-based metal electrode can be used in a flexible biosensor for wireless sensing.

**Figure 4.**
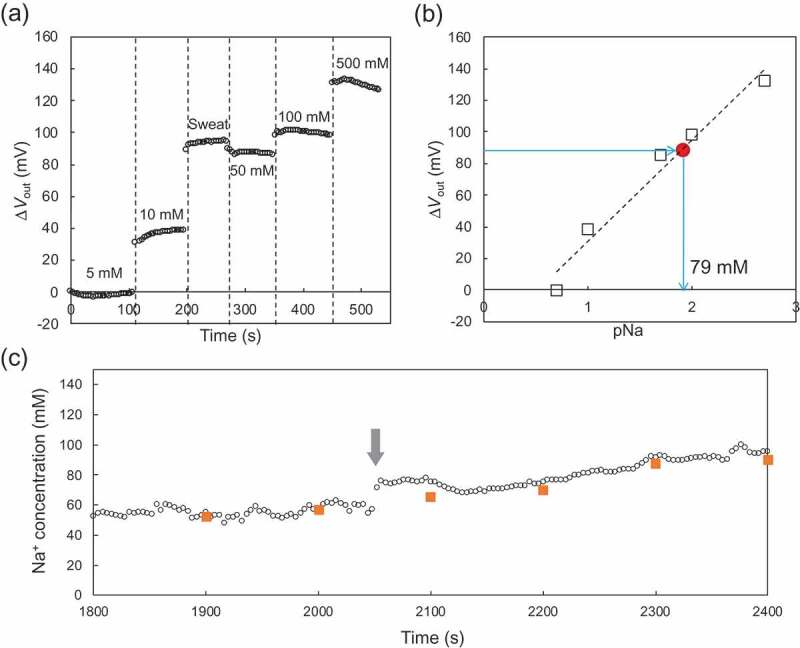
*In vitro* real-time monitoring of μ*V*_out_ for sweat sample using the paper-based Au electrode with the FPS-based Na^+^-sensitive membrane, which was rolled around the cylinder with the smallest radius of 6.5 mm. (a) μ*V*_out_ with changing Na^+^ concentrations from 5 mM to 500 mM in the buffer. The sweat sample was added after the measurement at 10 mM. (b) Calibration curve for pNa sensitivity based on (a). The Na^+^ concentration in the sweat sample was calculated from the calibration curve. (c) Changes in Na^+^ concentration determined by on-body measurement. The Na^+^ concentrations were calculated from the calibration curve obtained in (a) and (b). The Na^+^ concentration gradually increased after the time indicated by the arrow.

Moreover, the Na^+^ concentration in a sweat sample was evaluated using the paper-based Au electrode with the FPS-based Na^+^-sensitive membrane in a wireless and real-time manner, as shown in Figure 4. In this case, the modified paper-based metal electrode was rolled around the cylinder with the smallest radius of 6.5 mm. As shown in Figure 4(a), μ*V*_out_ increased with increasing Na^+^ concentrations from 5 mM to 500 mM in the buffer. When the sweat sample was added after the measurement at 10 mM, μ*V*_out_ remarkably increased. Then, μ*V*_out_ slightly decreased when 50 mM Na^+^ buffer was added onto the electrode surface. Moreover, Figure 4(b) shows the calibration curve for pNa sensitivity on the basis of the data obtained in Figure 4(a). The amount of the output signal for the sweat sample was obtained in Figure 4(a); therefore, the Na^+^ concentration in the sweat sample was calculated to be about 79 mM from the calibration curve (approximately 60 mV/pNa). In fact, the Na^+^ concentration in the sweat sample measured using the conventional Na^+^ sensor was about 74 mM. That is, the Na^+^ concentration in the sweat sample measured using the modified paper-based metal electrode indicated a good agreement with the expected concentration value. Moreover, the on-body measurement of Na^+^ concentration for sweat analysis was performed in a wireless and real-time manner. Figure 4(c) shows the changes in the Na^+^ concentration for the on-body measurement using the paper-based Au electrode with the FPS-based Na^+^-sensitive membrane. The Na^+^ concentrations were determined on the basis of the calibrated electrical signals shown in Figure 4(a,b). During the on-body measurement in the sauna, the Na^+^ concentration was stably monitored in a wireless and real-time manner, although it gradually increased from the point of time indicated by the arrow. This may have been due to a slight dehydration, under which condition, the Na^+^ concentration in sweat as well as in blood serum is known to increase[35]. Moreover, the sweat samples were collected from the forehead every 100 seconds to check the actual Na^+^ concentration using the conventional Na^+^ sensor, as indicated by the orange square plots in Figure 4(c). As a result, the Na^+^ concentrations obtained by this off-body measurement were in good agreement with those output by the on-body measurement. Therefore, we can expect that the Na^+^ concentration in the sweat sample can be measured using the paper-based Au electrode with the FPS-based Na^+^-sensitive membrane in a wireless and real-time manner, even if the modified paper-based metal electrode is put and bent around an arm. The on-body measurement of the change in the Na^+^ concentration in sweat may have future applications in the detection of dehydration during prolonged exercise. This is because the Na^+^ concentration increases in sweat as well as in blood serum[35].

### Microstructure

**Figure 5.**
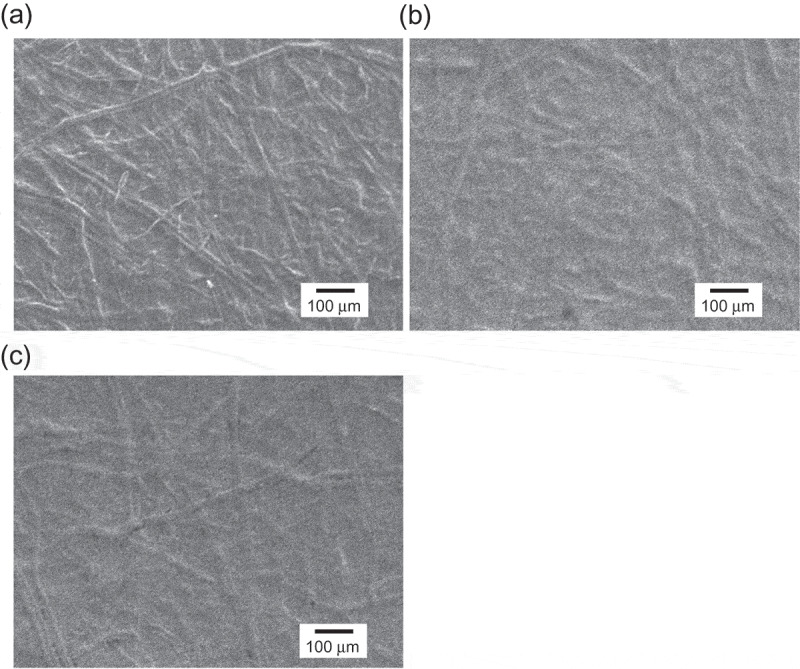
Scanning electron microscopy (SEM) images of paper-based Au electrodes (a) without modification, (b) with Ta_2_O_5_ modification, and (c) with Ta_2_O_5_ modification after bending around the cylinder (*r* = 6.5 mm).

Figure 5 shows SEM images of the original silicone-rubber-coated paper sheet and the paper-based Au electrode with the Ta_2_O_5_ film. The mesh structure of the original paper sheet was clearly observed (Figure 5(a)). In general, a mesh structure contributes to stress relaxation, thus preventing cracks from propagating in matrixes such as a paper sheet, enabling the paper sheet to be easily bent. Indeed, the mesh-patterned structure reduced tensile stress and hindered the propagation of cracks, as shown in a previous work[36]. Such meshes allow solutions to penetrate into paper sheets, but the paper sheets used in this study were coated with the silicone rubber; therefore, although the permeability of solutions into the paper sheets was poor, the sputtering of the Au/Cr thin film would have sufficiently coated the silicone rubber surface without mesh holes but with roughness at the micro scale. Furthermore, the complex microstructure of the paper sheets was transferred to the paper-based Au electrode with the Ta_2_O_5_ film sputtered on the silicone rubber surface (Figure 5(b)), and then the morphology of Ta_2_O_5_ film surface hardly changed owing to its micro scale roughness. This roughness depended on the mesh structure of paper even when the paper sheet electrode was rolled around the cylinder with the smallest radius of 6.5 mm (Figure 5(c)). This is why sufficient detection sensitivities of the paper-based metal electrodes were maintained upon bending, as shown in Figure 3.

Moreover, the thickness of the FPS-based Na^+^-sensitive membrane was measured using a 3D laser scanning confocal microscope (VK-X1000) to be about 114 μm, as shown in Figure S2a. In this case, the pNa sensitivity was obtained to be in the range of 50–60 mV/pNa, which is near the Nernstian response, as shown in Figures 3 and 4. Moreover, when the mixed solvent for the plasticizer-free Na^+^-sensitive membrane was diluted 50 times with THF, the thickness of the FPS-based Na^+^-sensitive membrane was measured using the laser microscope to be about 40 μm (Figure S2b). Regardless of the membrane thickness, the pNa sensitivity was maintained in the range of 50–60 mV/pNa in this study (data not shown).

Biocompatibility. In this study, the Ta_2_O_5_ film and the plasticizer-free FPS-based ISM on the paper-based Au electrodes were in direct contact with the sample solutions and made up the sensing surface. That is, these membranes will be in direct contact with a body to detect analytes in biological solutions such as sweat. In a previous study, the activities of mouse embryos on the Ta_2_O_5_ gate insulator of FET biosensors were continuously and safely monitored for about one week[22]. In particular, the mouse embryos cultured on the Ta_2_O_5_ film were transplanted to the recipient mice and the birth rate was almost equal to that for mouse embryos cultured on conventional cell culture dishes. Other living cells were also measured on Ta_2_O_5_-gate FET biosensors for a few days and no cytotoxicity was observed. Therefore, Ta_2_O_5_ films provide sufficient biocompatibility for use in wearable biosensors. Also, it was previously shown that no cytotoxicity was found for a plasticizer-free FPS-based ISM with calix[4]arene as an ionophore, although a PVC-based ISM showed cytotoxicity owing to the leaching of the plasticizer into sample solutions[28]. Considering the mobility and dispersibility of the ionophore in the membrane without a plasticizer, a matrix material that has a glass transition temperature below room temperature and polarity should be selected. In addition, it is desirable that the dielectric constant of the polymer used is in the range of 4–15 so as not to increase the membrane resistance, for example, FPS [37–39]. Thus, an FPS-based ion-sensitive membrane without a plasticizer has been utilized as a matrix for biocompatible Na^+^-sensitive sensors. Considering the above, the chemical modifications of the paper-based Au electrodes in this study can ensure noncytotoxic wearable biosensing.

## Conclusions

In this study, we developed paper-based metal electrodes with functional biointerfaces for use in wearable and nontoxic biosensors to monitor ions and biomolecules in a wireless manner, focusing on the detection of hydrogen ions and sodium ions as target analytes. The paper sheets thinly coated with silicone rubber showed flexibility owing to their meshed structure, even when various biocompatible functional membranes were deposited on them. In particular, the electrical stability, responsivity, and sensitivity of the modified paper-based Au electrodes were maintained over several weeks, even when they were bent to a radius of curvature in the range of 6.5 to 25 mm. Moreover, the Na^+^ concentration in the sweat sample was evaluated using the paper-based Au electrode with the FPS-based Na^+^-sensitive membrane in a real-time manner, even when the modified paper-based Au electrode was rolled around the cylinder with the smallest radius of 6.5 mm. Simultaneously, our newly developed miniaturized wireless device showed high performance in electrical measurement, assuming its use in a flexible body patch biosensor. Thus, a platform based on the paper-based metal electrode with flexibility and biocompatibility is suitable for wearable wireless biosensing. In the next plan, the paper-based metal electrodes may be fabricated in a soft microfluidic system with specific design strategies for inlet/outlet dimensions, where sweat samples would be not only collected but also discharged, as reported in a previous paper[40]. This is because the effect of residual ions from a previous measurement on electrical signals of the next measurement should be considered to continuously and precisely monitor the changes in the ion concentration in sweat samples in future studies.
